# Inflammatory Dendritic Cells Contribute to Regulate the Immune Response in Sickle Cell Disease

**DOI:** 10.3389/fimmu.2020.617962

**Published:** 2021-02-04

**Authors:** Renata Sesti-Costa, Marina Dorigatti Borges, Carolina Lanaro, Dulcinéia Martins de Albuquerque, Sara Terezinha Olalla Saad, Fernando Ferreira Costa

**Affiliations:** Hematology and Hemotherapy Center, University of Campinas, UNICAMP, Campinas, Brazil

**Keywords:** dendritic cell, sickle cell disease, heme oxygenase, inflammation, monocyte

## Abstract

Sickle cell disease (SCD), one of the most common hemoglobinopathies worldwide, is characterized by a chronic inflammatory component, with systemic release of inflammatory cytokines, due to hemolysis and vaso-occlusive processes. Patients with SCD demonstrate dysfunctional T and B lymphocyte responses, and they are more susceptible to infection. Although dendritic cells (DCs) are the main component responsible for activating and polarizing lymphocytic function, and are able to produce pro-inflammatory cytokines found in the serum of patients with SCD, minimal studies have thus far been devoted to these cells. In the present study, we identified the subpopulations of circulating DCs in patients with SCD, and found that the bloodstream of the patients showed higher numbers and percentages of DCs than that of healthy individuals. Among all the main DCs subsets, inflammatory DCs (CD14^+^ DCs) were responsible for this rise and correlated with higher reticulocyte count. The patients had more activated monocyte-derived DCs (mo-DCs), which produced MCP-1, IL-6, and IL-8 in culture. We found that a CD14^+^ mo-DC subset present in culture from some of the patients was the more activated subset and was mainly responsible for cytokine production, and this subset was also responsible for IL-17 production in co-culture with T lymphocytes. Finally, we suggest an involvement of heme oxygenase in the upregulation of CD14 in mo-DCs from the patients, indicating a potential mechanism for inducing inflammatory DC differentiation from circulating monocytes in the patients, which correlated with inflammatory cytokine production, T lymphocyte response skewing, and reticulocyte count.

## Introduction

Sickle cell disease (SCD) is a condition that affects millions of people worldwide. It is caused by a mutation in the β-globin gene that results in the replacement of a single glutamic acid with valine. As a consequence, an abnormal hemoglobin, HbS, is produced, which polymerizes under deoxygenation conditions. HbS polymerization causes changes in the shape and physical properties of erythrocytes, resulting in hemolytic anemia and the occlusion of small blood vessels (1, 2). Such crises are spontaneous and recurrent complications, in which microvascular infarction leads to episodes of extreme pain, multiple organ dysfunction, and infection (3–5). The vaso-occlusion process that occurs in patients with SCD is a complex phenomenon that involves the aggregation of erythrocytes, which interact with the endothelium and other blood cells, decreased nitric oxide (NO) bioavailability, oxidative stress, and the release of inflammatory cytokines into the bloodstream, such as TNF-α, IL-6, and IL-8 (6–8).

Patients with SCD are more susceptible to infection due to splenic hypofunction and impaired immune function (9–11). In addition to the deficiency described in innate immunity, including insufficient opsonization and phagocytosis of bacteria (12, 13), evidence of T and B lymphocyte dysfunction in SCD has also been reported in both patients and animal models. These changes include reduction in the proportion of circulating CD4^+^ and CD8^+^ T cells (14), defects in regulatory T cells (Tregs) (15), the polarization of cells to a T helper (Th)2 profile (16), and the loss of immunoglobulin (Ig)M-secreting memory B lymphocytes (17).

Dendritic cells (DCs) are the main cells responsible for adaptive immune response activation, polarization, and regulation. They are activated by both the pathogen- and damage-associated molecular patterns, which can be released by tissue necrosis or hemolysis (18–20), which are frequent occurrences in patients with SCD. Changes in DC activation or function may, therefore, be responsible for previously observed dysfunction in the T lymphocyte response. DCs can produce pro- or anti-inflammatory mediators depending on the tissue, the situation, and the cell subpopulation, as DCs form a heterogeneous population of cells with different functions. There are four main subsets of DCs, which share some roles, such as presenting antigens to lymphocytes, but they also have unique functions. Conventional type 1 DCs (cDC1) express the CD141 and Clec9a markers and are mainly responsible for CD8^+^ T lymphocyte activation and for polarizing the response to the Th1 profile. Conventional type 2 DCs (cDC2) express CD1c and Clec4a4 and are involved in lymphocyte differentiation to Th2 and Th17 profiles. Plasmacytoid DCs (pDCs), in turn, express the IL-3 receptor, CD123, and BDCA2, and produce large amounts of type I IFN. Finally, inflammatory DCs are derived from circulating monocytes during inflammation; they produce pro-inflammatory cytokines and are difficult to distinguish from cDCs2 (18, 21–23). Depending on the activation pattern and expression of inhibitory molecules, DCs are also essential in Treg differentiation, thus participating in the regulation of the immune response. The development of DCs and their differentiation to a specific cell subtype depends on the cytokines released in the microenvironment (24, 25), and the bloodstream of patients with SCD contains greater amounts of GM-CSF and IL-3, which are determinants in the development of cDCs and pDCs, respectively (8, 26). Nevertheless, the landscape of DC subsets in SCD remains unknown.

In the present study, we identified the subsets of circulating DCs in patients with SCD at steady state and showed that inflammatory DCs are elevated in the patients’ bloodstream, which correlated with hemolysis and IL-17–producing lymphocytes. Monocyte-derived DCs from patients produced inflammatory cytokines and skewed T lymphocyte responses towards a Th17 profile. Moreover, the inflammatory DCs arose from the patients’ monocytes in a heme oxygenase (HO)–dependent pathway. The present data provide new information on the initiation of the immune response in SCD, which may be related to susceptibility to infection and the sustained inflammatory component of the disease.

## Methods

### Human Samples

Patients with SCD (n = 47) and healthy controls (n = 46) aged 20 to 59 years were enrolled in the present study. Participants with HbSS or HbSβ^0^ genotypes were included as patients with SCD, and hemoglobin patterns were confirmed by high-performance liquid chromatography (HPLC) (Bio-Rad) and DNA sequence analysis. Patients who had received blood transfusions in the past 3 months, in vaso-occlusive crises and with apparent infection were excluded from the study. No patient was being treated with antibiotics or corticosteroids, and all patients were under treatment with folic acid, calcium, and vitamin D. Moreover, most patients were under treatment with hydroxyurea. Blood samples were obtained from the participants during regular consultation at the Unicamp Hematology and Hemotherapy Center, São Paulo, Brazil. Complete blood counts with reticulocyte counts were performed on blood collected with EDTA in a hematology analyzer (Beckman Coulter). The study was approved by the Unicamp Human Research Ethics Committee (protocol number CAAE: 85061318.0.0000.5404). All patients and controls had agreed to participate and had signed informed consent forms.

### Separation of Peripheral Blood Mononuclear Cells (PBMCs) and Isolation of Monocytes and T Lymphocytes

Blood was collected from all participants in heparin-coated tubes. The fresh blood was diluted in phosphate-buffered saline (PBS), and PBMCs were obtained by density gradient centrifugation on Ficoll-Hypaque (GE Healthcare) at 400*g* for 30 min at room temperature. The mononuclear leukocyte layer was separated and washed. The pellet with the remaining aggregated red blood cells was lysed with lysis buffer and washed with PBS. PBMCs were resuspended in RPMI 1640 medium (Gibco) supplemented with 10% fetal bovine serum, penicillin and streptomycin, and l-glutamine. Monocytes were isolated from the PBMCs by anti-CD14-coated magnetic beads (Miltenyi Biotec), and T lymphocytes were isolated using human a pan-T cell isolation kit (Miltenyi Biotec) according to the manufacturer instructions.

### Cell Phenotyping by Flow Cytometry

PBMCs were incubated in staining buffer (PBS; ACD; 10% BSA) and stained with monoclonal antibodies conjugated to PE, PE-Cy7, FITC, APC, APC-Cy7, PerCP-Cy5, BV451, BV605, or BV650 for 30 min at 4°C. DCs were stained using antibodies against CD3, CD19, CD56, CD14, HLA-DR, CD1c, CD141, CD123, and CD135 (BD Biosciences and BioLegend). The absolute number of DCs per µl of blood was calculated by multiplying the percentages by the complete blood leukocyte count. Monocytes were analyzed using antibodies against CD14 and CD16. Cells were washed and evaluated by flow cytometry (Cytoflex, Beckman Counter). For analyzing Tregs, surface marker staining was performed as described above with anti-CD3 and anti-CD4 antibodies, and then the cells were washed, fixed, and permeabilized with a Foxp3 Cytofix/Cytoperm kit (BD Biosciences) according to the manufacturer’s instructions, and stained with anti-FOXP3 antibody for an additional 30 min at 4°C. The cells were washed before acquisition in flow cytometry. To evaluate intracellular cytokines in lymphocytes, PBMCs were stimulated with 500 μg/ml PMA and 50 μg/ml ionomycin in the presence of GolgiStop (BD Biosciences) for 4 h, then stained with conjugated antibodies against CD3, CD4, and CD8. The cells were washed, fixed, and permeabilized using a Cytofix/Cytoperm kit (BD Biosciences) using the manufacturer’s instructions. Then, the cells were incubated with monoclonal antibodies against IFN-γ, IL-10, or IL-17 for 30 min at 4°C, washed, and evaluated by flow cytometry. Data were analyzed using FlowJo (BD Biosciences).

### Generation of Monocyte-Derived DCs

DCs were differentiated from isolated monocytes in culture by incubation in RPMI 1640 medium (Gibco) supplemented with 10% fetal bovine serum, L-glutamine, penicillin, streptomycin, and containing 20 ng/ml GM-CSF and 20 ng/ml IL-4 (R&D Systems) for 7 days. The medium containing the cytokines was replenished on the third day. In some experiments, monocytes were incubated with the HO-1 inhibitor SnPP (tin protoporphyrin IX dichloride, R&D Systems; 50 µM) for 1 h before and during DC differentiation. The cells were analyzed by flow cytometry, and the cytokine levels in the supernatant were quantified. Monocyte-derived DCs (mo-DCs) were stained using antibodies against CD14, CD209, CD1c, CD86, HLA-DR, and CD83 (BD Biosciences and BioLegend). In other experiments, the cells were counted and incubated with T lymphocytes to perform co-culture.

### Co-culture of DCs and T Lymphocytes

The mo-DCs were counted, and 5 × 10^4^ cells were plated in 96-well culture plates. After 24 h, T lymphocytes were isolated from the PBMCs from the healthy controls and stained with 2.5 μM CFSE (carboxyfluorescein succinimidyl ester) for 15 min at room temperature. T lymphocytes (5 × 10^5^) were incubated with mo-DCs for an additional 5 days. Lymphocyte proliferation was evaluated by flow cytometry based on CFSE dilution in the CD4 or CD8 gates after staining with antibodies against CD4 and CD8. The co-culture supernatant was harvested and used to measure cytokine levels.

### Cytokine Measurement

The cytokines IL-1β, IFN-α2, IFN-γ, TNF-α, MCP-1, IL-6, IL-8, IL-10, IL-12p70, IL-17A, IL-18, IL-23, and IL-33 were measured in the supernatant of DCs and co-cultures using the LEGENDplex Human Inflammation Panel 1 (BioLegend) according to the manufacturer’s instructions. The acquisition was performed by flow cytometry (Cytoflex, Beckman Coulter), and the results were analyzed by LEGENDplex software.

### Quantitative Real-Time PCR

Total RNA was extracted from monocytes using an RNeasy Mini Kit (Qiagen), and genomic DNA was digested with DNase I (Fermentas). Reverse transcription was performed using a RevertAid H Minus First Strand cDNA Synthesis Kit (Thermo Scientific), and *Hmox1* expression was analyzed using SYBR Green PCR master mix (Applied Biosystems). The following primers were used: *Hmox1* forward: 5′-GACGGCTTCAAGCTGGTGAT-3′ and reverse: 5′-GTTGCGCTCAATCTCCTCCT-3′; *Rplpo* (ribosomal protein lateral stalk subunit P0) forward: 5′-GGAAGGCTGTGGTGCTGATG-3′ and reverse 5′-GAGGCAGCAGTTTCTCCAGAG-3′.

### Statistical Analyses

The mean ± SEM values are presented in the graphs, and the pair of groups were compared using the Mann-Whitney test for non-Gaussian distributed data. Pairs of cells sorted from the culture of the same patient were analyzed using paired Student’s *t*-tests. *P* values < 0.05 were considered statistically significant. All data were analyzed using Prism 5.1 software (GraphPad).

## Results

### Patients With SCD Had Increased Total DCs and Altered Ratio of DC Subsets

**Figure 1:**
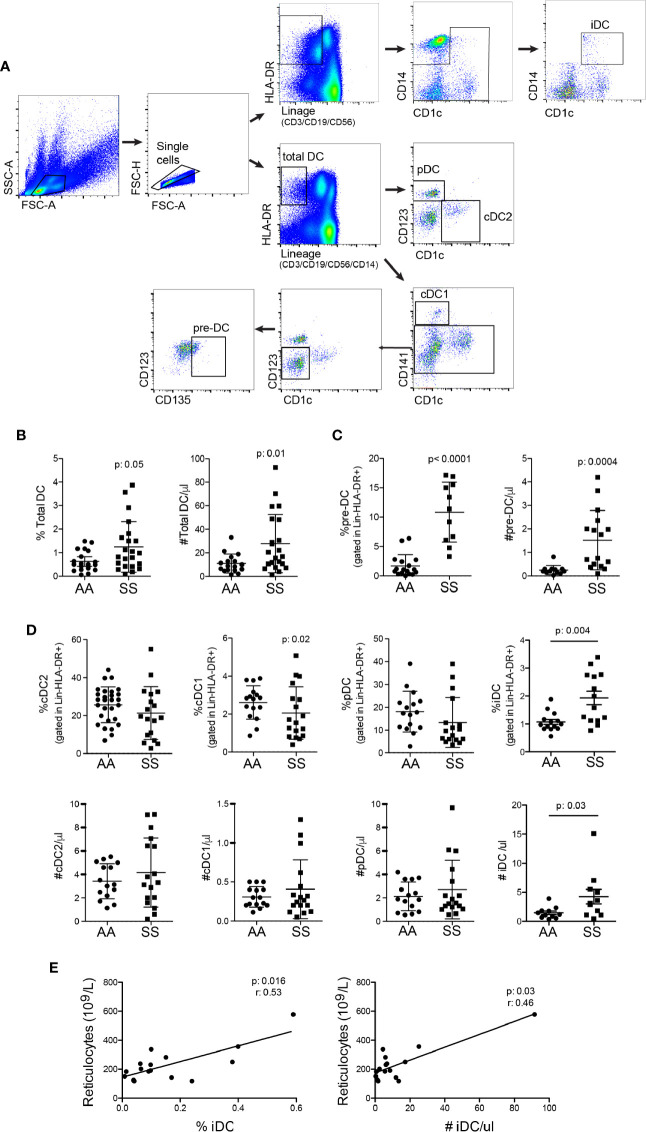
Patients with SCD have an increased number of total DCs in the bloodstream and distinct proportions of DC subsets. The different DC subsets were identified in the blood from the patients (SS: hemoglobin S) and controls (AA: hemoglobin A) by flow cytometry. **(A)** The gating strategy used to identify total DCs, namely, lineage^−^ (CD3/CD19/CD56/CD14) HLA-DR^+^; pre-DCs (lineage^−^HLA-DR^+^CD1c^−^CD141^−^CD123^−^CD135^+^); cDC1 (lineage^−^HLA-DR^+^CD1c^−^CD141^+^CD123^−^); cDC2 (lineage^−^HLA-DR^+^CD1c^+^CD141^−^CD123^−^); pDC (lineage^−^HLA-DR^+^CD1c^−^CD141^−^CD123^+^), and iDC is lineage^−^ (CD3/CD19/CD56), HLA-DR^+^CD1c^+^CD14^+^. **(B)** The percentage of total DCs in PBMCs and their absolute number per µl blood. **(C)** The percentage of pre-DCs in total DCs and their absolute number per µl blood. **(D)** The percentage within total DCs (top panel) and the absolute number per µl (bottom panel) cDC2, cDC1, pDC, and iDC. N: AA = 20; SS = 22. *P*-values were obtained using the Mann-Whitney test. **(E)** Spearman correlation between %iDC (left panel) and #iDC (right panel) with circulating reticulocyte numbers.

To characterize the main DC subsets in the bloodstream of the patients, we isolated PBMCs from their blood and performed multicolor flow cytometry. The gating strategy used for identifying the DC population (**Figure 1A**), was to gate on lymphocyte and monocyte populations by forward scatter and side scatter plots, followed by gating in single cells. Next, total DCs were detected as negative for lineage markers (CD3/CD19/CD56/CD14) and HLA-DR^+^. Within the total DC population, additional gates were designed to distinguish the different DC subsets and pre-DC population. Pre-DCs were considered lineage^−^HLA-DR^+^CD1c^−^CD141^−^CD123^−^CD135^+^; cDC1 as lineage^−^HLA-DR^+^CD1c^−^CD141^+^CD123^−^; cDC2 as lineage^−^HLA-DR^+^CD1c^+^CD141^−^CD123^−^; and pDC as lineage^−^HLA-DR^+^CD1c^−^CD141^−^ CD123^+^ (19, 27, 28). A subset of DCs expressing CD14, henceforth referred to as inflammatory DCs (iDCs), was identified as negative for lineage markers (CD3/CD19/CD56) and were HLA-DR^+^CD1c^+^CD14^+^ (29).

The patients had total DCs increased in the blood (**Figure 1B**). The result was very heterogeneous among the patients, some of them had very high numbers of DCs, whereas others presented numbers similar to controls. The development of DCs from the bone marrow is possible to be, at least in part, responsible for this increase, as the percentage and total number of DC precursors were augmented in the patients’ blood (**Figure 1C**). Notably, the increased amount of DCs is unlikely to be only a result of leukocyte redistribution due to splenic hypofunction, as the percentage of DCs among white blood cells was also increased (**Figure 1B**). The discrimination of DC subsets revealed that the ratio of cDC1 was reduced, whereas that of iDC was increased in the patients relative to the controls. The percentages of cDC2 and pDC were similar between the groups (**Figure 1D**). iDC was the only subset with a significantly higher total number in the patients’ blood. None of the other three main subsets alone accounted for the increase in total DCs. Their numbers were slightly higher in the patients than in the controls, but the difference was not statistically significant (**Figure 1D**), suggesting that all subsets together and pre-DCs may contribute to the higher number of circulating DCs. Notably, both the percentage and total number of iDCs were significantly correlated with the number of reticulocytes in the blood (**Figure 1E**), a *bona fide* hemolysis parameter.

### Mo-DCs From Patients With SCD Showed an Activated Phenotype and Produced Inflammatory Cytokines

**Figure 2:**
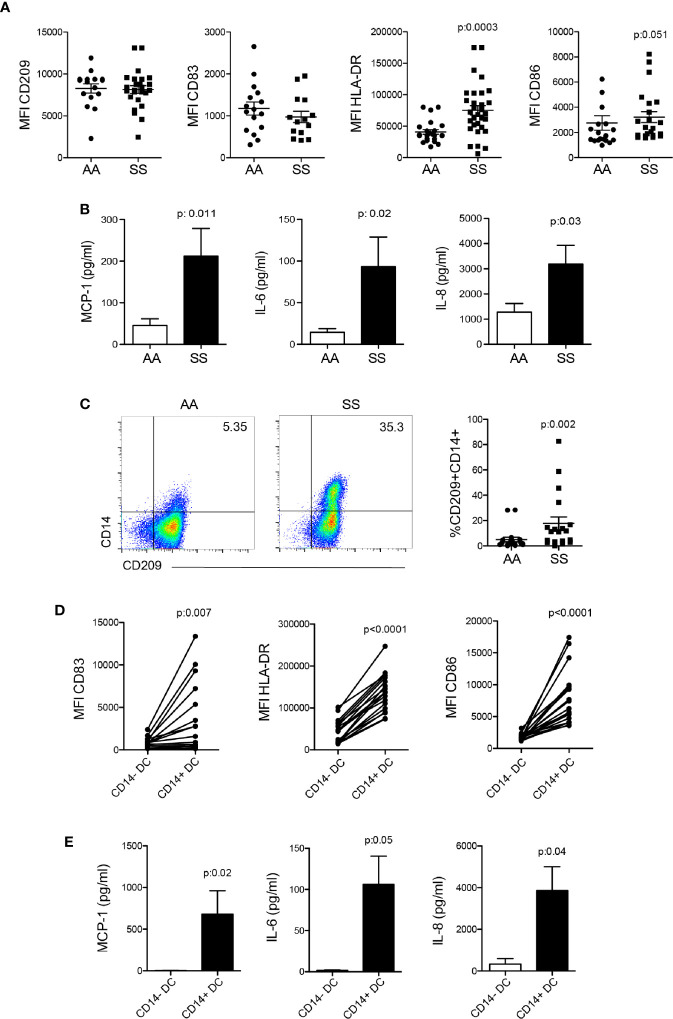
Activation profile and inflammatory cytokine production by mo-derived DCs from patients with SCD. Monocytes were isolated from the PBMCs of the patients (SS) and controls (AA) and cultured with 20 ng/ml GM-CSF and IL-4 for 6 to 7 days for DC differentiation. **(A)** Flow cytometry analysis of DC activation marker expression. **(B)** LEGENDplex measurement and flow cytometry acquisition of cytokine secretion in the supernatant after 24 h. **(C)** CD14 expression in CD209^+^HLA-DR^+^ DCs after differentiation. **(D)** Analysis of DC activation markers in the CD14^+^ and CD14^−^ DC subpopulations. *P*-values were obtained using the Mann-Whitney test. **(E)** CD14^+^ and CD14^−^ DCs from the same patient were sorted by flow cytometry and incubated for 24 h. Cytokines were measured in the supernatant by LEGENDplex. *P*-values were obtained using the paired Student’s *t*-test.

iDCs are cells mainly derived from activated monocytes present in the circulation. Thus, we characterized the DCs differentiated *in vitro* in the presence of GM-CSF and IL-4 from monocytes (mo-DCs) isolated from the PBMCs of the patients and the controls. More mo-DCs from the patients presented an activated phenotype, as shown in the higher expression of the presenting antigen molecule HLA-DR and the co-stimulator CD86. No difference was seen in the expression of the DC marker CD209 and the maturation molecule CD83 (**Figure 2A**). Interestingly, DCs from the patients produced two- to three-fold more MCP-1, IL-6, and IL-8 compared to the controls even with no *in vitro* stimulation (**Figure 2B**), indicating a potential role for these cells in monocyte and neutrophil recruitment to the inflammatory microenvironment. A more detailed analysis of mo-DCs revealed the expression of the monocyte marker CD14 in some patients (**Figure 2C**). These cells expressed the DC markers CD209 (**Figure 2C**) and CD1c, and were smaller than monocytes (data not shown), which characterized them as iDCs. Furthermore, CD14^+^ DCs showed higher expression of the DC maturation marker CD83, and the activation markers HLA-DR and CD86 than CD14^−^ DCs from the same patient (**Figure 2D**). Next, we sorted CD14^+^ DCs and CD14^−^ DCs and incubated them for 24 h without any additional stimulation. Surprisingly, the CD14^+^ DCs were among the mo-DCs responsible for the production of MCP-1, IL-6, and IL-8 in the culture supernatant (**Figure 2E**).

### Patients With SCD Had More Activated T Lymphocytes and More IL-17 Production

**Figure 3:**
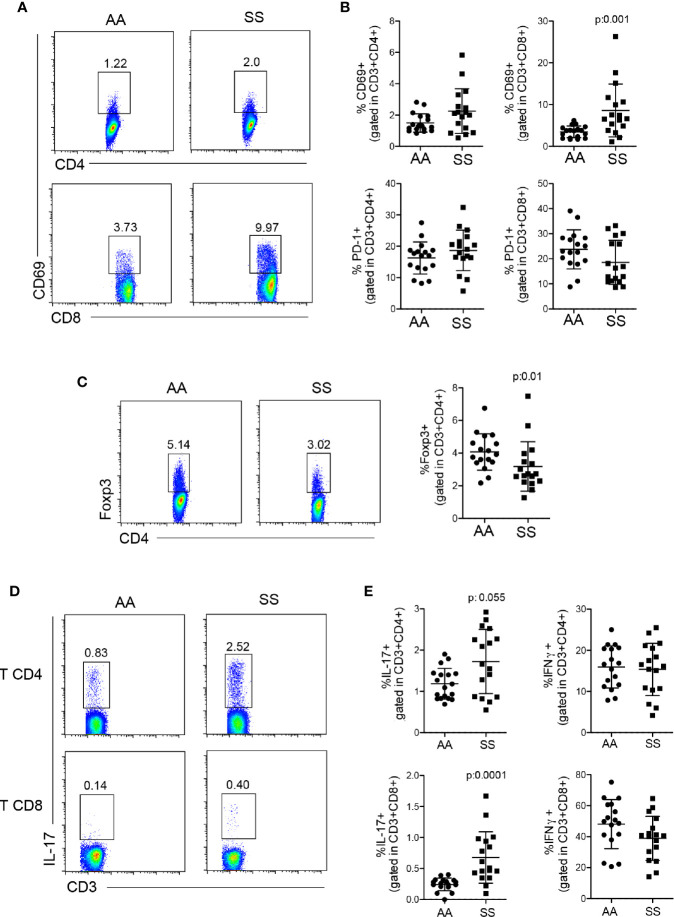
Profile of T lymphocyte response in patients with SCD. **(A, B)** Activation and exhaustion of CD4^+^ and CD8^+^ T lymphocytes were quantified by staining of PBMCs from the patients (SS) and controls (AA) with CD69 and PD-1, respectively, within CD3^+^CD4^+^ or CD3^+^CD8^+^ populations. **(A)** Representative dot plots of CD69. **(B)** The percentage of CD69 (top) and PD-1 (bottom)-stained cells within CD4^+^ (left) and CD8^+^ (right) T lymphocytes. **(C)** Tregs were identified in PBMCs by the percentage of FOXP3^+^CD4^+^ T lymphocytes. The left panel shows the representative dot plots; the right panel shows the percentage of Tregs in the patients (SS) and controls (AA). **(D, E)** PBMCs were stimulated *in vitro* with PMA and ionomycin in the presence of brefeldin for 4 h, then stained for intracellular cytokines. **(D)** Representative dot plots of IL-17. **(E)** The percentage of IL-17 (left) and IFN-γ (right)-producing CD4^+^ (top) and CD8^+^ (bottom) T lymphocytes. N: AA = 17; SS = 17. *P*-values were obtained using the Mann-Whitney test.

DCs are the major cells responsible for modulating lymphocyte function (20, 30, 31). Thus, the next step was to determine T cell responses in the circulation of this cohort of patients to associate them with the alterations in DC numbers and ratios. We observed a greater percentage of T lymphocytes from the patients as compared to that from the controls, especially CD8^+^ T lymphocytes, which presented a statistically significant difference in CD69 expression, a T cell activation marker (**Figures 3A, B**). Although SCD is a chronic inflammatory disease, the T lymphocytes did not present an exhaustion profile, as PD-1 expression by both CD4^+^ and CD8^+^ T cells was comparable to that of the controls (**Figure 3B**). Tregs are important cells involved in regulating exacerbated inflammation *via* several mechanisms (32). Compared with the controls, the patients had reduced Tregs, as demonstrated by the lower percentages of FOXP3^+^CD4^+^ T lymphocytes in their bloodstream (**Figure 3C**). Then, we stimulated PBMCs *in vitro* with PMA and ionomycin in the presence of brefeldin to analyze the profile of cytokines produced by the T cells. Both CD4^+^ and CD8^+^ T lymphocytes from the patients produced higher amounts of IL-17 (**Figures 3D, E**). No difference was seen in IFN-γ production by T lymphocytes in this cohort (**Figure 3E**), and IL-10 production by *in vitro* stimulation was very low and similar between the groups (data not shown). Altogether, these data suggest that the lower ratio of Tregs and the skewed T cell response to Th17 and Tc17 (IL-17–secreting CD8 T cells) profiles can cooperate for the inflammatory phenotype and altered adaptive immune response seen in the patients.

### Mo-Derived DCs From the Patient with SCD Stimulated T Lymphocyte Proliferation and IL-17 Production

**Figure 4:**
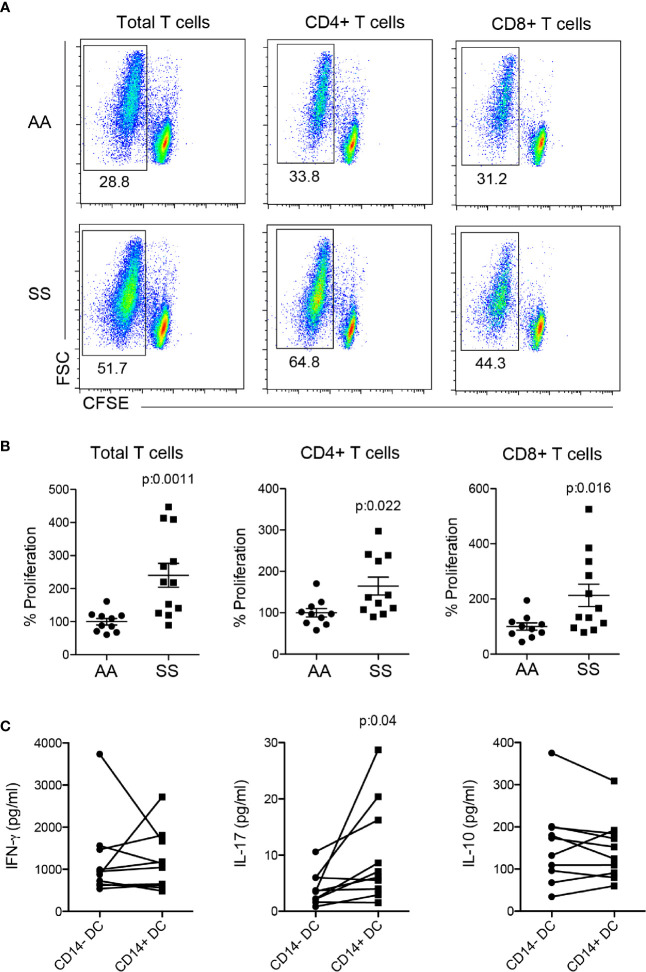
Co-culture of mo-derived DCs and T lymphocytes. Monocytes were isolated from PBMCs of the patients (SS) and controls (AA) and cultured with 20 ng/ml GM-CSF and IL-4 for 6–7 days for DC differentiation. Mo-DCs were co-cultured (1:10) with allogeneic T lymphocytes from healthy individuals; the T lymphocytes had been previously stained with CFSE for 5 days. **(A, B)** T lymphocyte proliferation was evaluated by CFSE dilution and CD4 and CD8 staining. **(A)** Representative dot plots and **(B)** graphs of the percentage of proliferation measured by CFSE^−^ gating. AA was considered 100%. *P*-values were obtained using a Student’s *t*-test. **(C)** CD14^+^ and CD14^−^ mo-DCs were sorted by flow cytometry before co-culture with T lymphocytes. Cytokines were measured in co-culture supernatant by flow cytometry. *P*-values were obtained using a paired Student’s *t*-test.

To address whether DCs are responsible for the T cell phenotype observed in the patients, we performed a co-culture assay of mo-DCs from the patients or controls with allogeneic T cells stained with CFSE. Mo-DCS from the patients induced higher-intensity proliferation of total T lymphocytes as compared to that of the controls. When the analysis was stratified in CD4^+^ and CD8^+^ T lymphocytes, we observed that both cells contributed to the higher proliferation of the T cell population (**Figures 4A, B**), showing that mo-DCs from the patients had additional capability for stimulating both CD4^+^ and CD8^+^ T cells, absent in those from the controls. Notably, when the sorted CD14^+^ and CD14^−^ DCs from the same patient were used in co-culture, the CD14^+^ DCs induced higher IL-17 production than their counterparts, whereas IFN-γ and IL-10 were induced with the same intensity (**Figure 4C**), indicating that CD14^+^ DCs are, at least in part, responsible for the Th17/Tc17 phenotype in patients with SCD.

### Upregulation of HO-1 by Monocytes Induced CD14^+^ DC Differentiation in Patients With SCD

**Figure 5:**
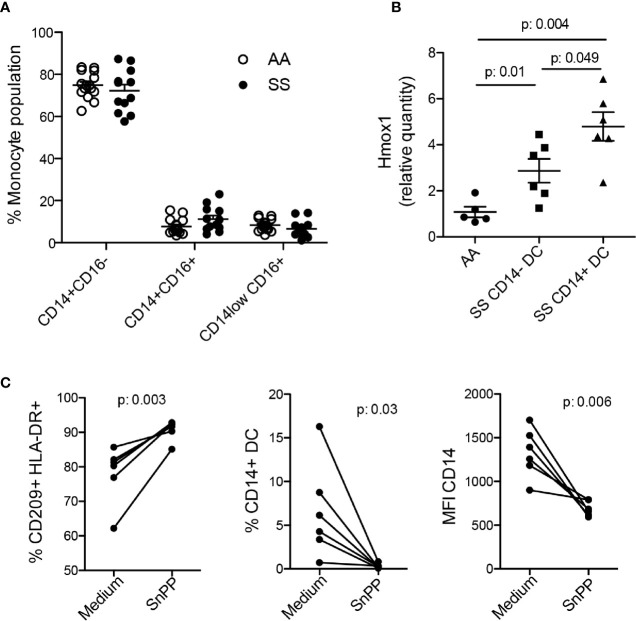
The role of HO-1 in iDC differentiation. **(A)** Phenotyping of monocyte subsets in the controls (AA) and patients (SS) by flow cytometry**. (B)** Monocytes were isolated from the PBMCs of the patients (SS) and controls (AA). A portion was cultured with 20 ng/ml GM-CSF and IL-4 for 6 to 7 days for DC differentiation and evaluation of CD14 expression. The other portion underwent RNA extraction for RT-PCR evaluation of HO-1 transcript expression (*Hmox1*). The graph shows data separately from patients with (SS CD14^+^ DC) or without (SS CD14^−^ DC) CD14^+^ DC differentiation. *P*-values were obtained using the Mann-Whitney test. **(C)** Monocytes isolated from the PBMCs of the patients were treated with SnPP (50 µM) for 1 h, then induced to differentiate into DCs. The percentage and mean fluorescence intensity (MFI) were measured by flow cytometry. *P*-values were obtained using a paired Student’s *t*-test.

In an attempt to unveil the mechanisms by which monocytes from some patients differentiate into CD14^+^ DCs, we evaluated the distribution of monocyte subtypes in the bloodstream. In the present cohort, we did not find any difference in the monocyte subtype ratio between the patients and the controls (**Figure 5A**), suggesting no role for subset-specific monocytes in iDC differentiation and no correlation of a monocyte subset with DCs compartment changes. Monocytes from patients with SCD are constantly exposed to heme released during hemolysis, and there is evidence that they can upregulate HO-1, which metabolizes heme into CO (carbon monoxide), Fe (iron), and biliverdin (33, 34). Thus, we evaluated HO-1 expression by monocytes, and found that monocytes from the patients overall demonstrated upregulation of HO-1 compared to that from the controls. Notably, monocytes from the patients that differentiated *in vitro* into CD14^+^ DCs (SS CD14^+^ DC) showed even higher upregulation of HO-1 expression than the monocytes that differentiated only into CD14^−^ DCs (SS CD14^−^ DC) (**Figure 5B**). Hence, to reveal the role of HO-1 in CD14^+^ DC differentiation, we treated monocytes from the patients with the HO-1 inhibitor, SnPP, for 1 h before and during *in vitro* DC differentiation. SnPP treatment increased the percentage of DCs differentiated in culture, as seem by HLA-DR^+^ CD209^+^ cells ratio (**Figure 5C**). However, the treatment decreased both the percentage and mean fluorescence of CD14 on mo-DCs (**Figure 5C**), indicating a previously unknown mechanism of HO-1 in iDC differentiation in patients with SCD.

Altogether, the data show that the number and ratio of DCs are increased in the circulation of patients with SCD and that the amount of iDCs correlates with hemolysis. DCs from patients with SCD, especially CD14^+^ DCs, show an activated phenotype and produce inflammatory cytokines seen to be responsible for monocyte and neutrophil recruitment, indicating that they may cooperate in the inflammatory milieu. In addition, they demonstrated an association with T lymphocyte activation and IL-17 production. Finally, we found that the differentiation of iDCs from monocytes may occur through an HO-1–dependent pathway.

## Discussion

Despite exceptional advances in the last decade in SCD therapy, including new drugs, bone marrow transplantation, and gene therapy (35–37), both basic and clinical research is ongoing to obtain greater knowledge of the cells and mediators involved in the disease, and to develop new and better therapies that improve patient quality of life.

DCs are key players in the initiation of the immune response to pathogens and also of tolerance to self, microbiota, and dietary antigens (20, 38). Changes in DC function can result in prevention of the fight against infection or in the development of autoimmune and inflammatory diseases (39–41). The role of DCs and their specific subsets in the chronic inflammatory component or adaptive immune dysfunction of SCD remains elusive. In the present work, we show for the first time that circulating DCs are increased and that the ratio of DC subsets is altered in patients with SCD. The higher numbers of circulating pre-DCs may indicate intensification in DC development from the bone marrow. In addition, as iDCs can arise from circulating monocytes, they are likely to be another source of DC expansion in the bloodstream, as our data show higher numbers of this DC subpopulation. We also observed a reduction in the circulating cDC1 in the patients. It remains uncertain whether the development of this subpopulation is reduced or whether they are being activated and subsequently migrate to the lymph nodes, where they prime T lymphocytes. These hypotheses are difficult to address in humans; nevertheless, they warrant future investigation. Notably, we found a heterogeneity among the patients regarding circulating DCs ratios and a correlation between iDC percentage and number with reticulocyte counts, suggesting that changes in DCs compartment may be present only in patients with certain degree of disease severity. Although we could not establish a clear cause and effect relationship, we observed that the percentage of DCs was also increased in the patients, suggesting a skewing towards DC development rather than a general leukocyte increase or redistribution.

Here, *in vitro* differentiation of monocytes into DCs revealed that the patients’ monocytes could derive more activated and inflammatory iDCs, as they produced MCP-1, IL-6, and IL-8 even without stimulation. No difference was observed in the DC or maturation markers CD209 and CD83, respectively, which was also shown in an investigation of mo-DCs in the context of SCD alloimmunization (42). That study also suggests that mo-DCs from patients with SCD produce more inflammatory cytokines, as the authors showed that SCD mo-DCs produced more IL-12 than those from healthy individuals after stimulation with LPS, LPS+IFN-γ, or R848. Additionally, some of the patients’ mo-DCs expressed the monocyte marker CD14. Despite this, they had the DC phenotype, expressed CD209 and CD1c, and were smaller than monocytes. CD14^+^ mo-DCs are more mature and activated than CD14^−^ mo-DCs, and are responsible for inflammatory cytokine production. Patients with SCD present an expansion of monocyte and neutrophil counts, which interact with the activated endothelium, cooperating in vaso-occlusion (43, 44). Although our *in vitro* system did not allow us to estimate the contribution of DCs to inflammatory cytokine production *in vivo* in the bloodstream compared to other cells, the present data indicate that the DCs of patients with SCD, especially CD14^+^ DCs, produce these cytokines, which may be associated with their systemic amounts and the migration of monocytes and neutrophils to DC sites. Thus, it is important to determine whether DCs are present and participate in the vaso-occlusion process.

As the T lymphocyte response in patients with SCD varies according to the cohort evaluated and the parameter analyzed, we assessed T cells in the present cohort to associate to the DC phenotype. We found that the patients had more CD4^+^ and CD8^+^ T lymphocytes and higher IL-17 production. Several studies have shown the involvement of IL-17 in neutrophil activation and recruitment and in the development of autoimmune and inflammatory disease (45–48); thus, IL-17 may participate in the inflammatory process in patients with SCD and can alter their immune response to a subsequent infection. Depending on the context, previous studies have shown variable results for the production of IFN-γ and Th1-biased responses in patients with SCD (16, 49, 50). In our cohort, IFN-γ production by both CD4^+^ and CD8^+^ T cells was similar between the patients and the controls. In addition, the patients showed a reduced percentage of Tregs, which may contribute to the inflammatory state. We demonstrate the role of DCs in some T lymphocyte responses in the patients. Our results show that the DCs of patients with SCD are more capable of inducing both CD4^+^ and CD8^+^ T cell proliferation, and that CD14^+^ DCs stimulated higher IL-17 production in co-culture than CD14^−^ DCs. The mechanisms by which CD14^+^ DCs induce IL-17 production are still unknown. IL-6, IL-23, and TGF-β production by DCs polarizes T lymphocytes to a Th17 profile (51). In our co-culture system, although we observed higher IL-6 production by the patients’ DCs, especially CD14^+^ DCs, IL-23 was undetectable in the supernatant of unstimulated DCs (data not shown), suggesting no participation of these cytokines in the process.

Although other groups have found changes in monocyte subpopulations in patients with SCD (52), our patient cohort did not show any difference in the monocyte subpopulations compared with the controls, which indicates that no specific monocyte subset is responsible for CD14^+^ DC differentiation. The difference between previous data and ours may be because, for most of the experiments, all patients from our cohort were under hydroxyurea treatment, which may directly or indirectly reverse the changes in monocyte subset ratio caused by the disease, as previously shown (53). Due to changes in membrane lesion secondary to HbS polymerization into erythrocytes, SCD is characterized by a percentage of intravascular hemolysis (54), which results in the release of free hemoglobin and free heme in circulation. Free heme can modulate monocyte and mo-DC functions in SCD (42, 55) and in several other diseases (56) through the TLR4 or HO-1 pathways. In our work, we found that HO-1 expression in the patients’ monocytes was upregulated compared to that from the controls, as previously reported (33). Surprisingly, patients whose monocytes could differentiate into CD14^+^ DCs in culture expressed even more HO-1 than the patients whose monocytes differentiated only into CD14^−^ DCs. The inhibition of HO-1 enzymatic activity in the patients’ monocytes prevented CD14 expression in the posteriorly derived DCs, indicating a possible mechanism by which the monocytes from some patients differentiate into these iDCs. Further investigation is still need to confirm this possibility and would provide more important information about the stimulus responsible for HO-1 upregulation, the signaling pathways downstream to HO-1 involved in DCs differentiation, and the changes in DCs functions observed. In summary, the present study reports novel findings regarding the role of DCs in SCD and provides new insights into the chronic inflammation and the immune dysfunction observed in patients with SCD.

## Data Availability Statement

The raw data supporting the conclusions of this article will be made available by the authors, without undue reservation.

## Ethics Statement

The studies involving human participants were reviewed and approved by Unicamp Human Research Ethics Committee (protocol number CAAE: 85061318.0.0000.5404). The patients/participants provided their written informed consent to participate in this study.

## Author Contributions

RS-C and FFC designed the experiments and wrote the manuscript. RS-C and MDB performed the experiments. CL and DMA collaborated in some experiments. STOS and FFC assisted the patients. FFC supervised the work. All authors contributed to the article and approved the submitted version.

## Conflict of Interest

The authors declare that the research was conducted in the absence of any commercial or financial relationships that could be construed as a potential conflict of interest.
